# Selective Separation of 1-Butanol from Aqueous Solution through Pervaporation Using PTSMP-Silica Nano Hybrid Membrane

**DOI:** 10.3390/membranes10040055

**Published:** 2020-03-26

**Authors:** VSSL Prasad Talluri, Aiym Tleuova, Seyedmehdi Hosseini, Ondrej Vopicka

**Affiliations:** 1 Department of Biotechnology, University of Chemistry and Technology, Technicka 5, 166 28 Prague, Czech Republic; 2 Chemical Engineering Department, University of Chemistry and Technology, Technická 5, 166 28 Praha 6, Czech Republic; 3 Department of Metals and Corrosion Engineering, University of Chemistry and Technology Prague, 166 28 Prague, Czech Republic; 4 Department of Physical Chemistry, University of Chemistry and Technology, Technická 5, 166 28 Prague 6, Czech Republic

**Keywords:** PTMSP, Silica, 1-butanol, water, pervaporation

## Abstract

In this work, a poly(1-trimethylsilyl-1-propyne) (PTMSP) mixed-matrix membrane was fabricated for the selective removal of 1-butanol from aqueous solutions through pervaporation. Silica nanoparticles (SNPs), which were surface-modified with surfactant hexadecyltrimethylammonium bromide (CTAB), were incorporated into the structure of the membrane. The modified membrane was characterized by thermogravimetry-differential scanning calorimetry (TG-DSC), contact angle measurements, and scanning electron microscope (SEM) analysis. It was found that the surface hydrophobicity of the membrane was improved when compared to neat PTMSP by contact angle measurement. It was confirmed by SEM analysis that a uniform distribution of surface-modified SNPs throughout the PTMSP membrane was achieved. The thermogravimetric analysis detected the thermal degradation of the modified PTMSP at 370 °C, which is comparable to neat PTMSP. The pervaporation measurements showed a maximum separation factor of 126 at 63 °C for 1.5 w/w% 1-butanol in the feed. The maximum total flux of approximately 1.74 mg·cm^−2^·min^−1^ was observed with the highest inspected temperature of 63 °C and at the 1-butanol concentration in the feed 4.5 w/w%. The pervaporation transients showed that the addition of the surface-modified SNPs significantly enhanced the diffusivity of 1-butanol in the composite compared to the neat PTMSP membrane. This improvement was attributed to the influence of the well-dispersed SNPs in the PTMSP matrix, which introduced an additional path for diffusivity.

## 1. Introduction

Butanol is an ecological and practically non-toxic solvent and an important chemical feedstock, which has been extensively used in many industries [1]. Butanol is also considered as a possible replacement for fossil fuels when produced from acetone butanol ethanol (ABE) fermentation [2]. This compound, which is also known as 1-butanol or *n*-butanol or *n*-butyl alcohol (biobutanol when produced biologically), is a four-carbon straight-chain alcohol-based compound, which occurs as a colorless liquid with a distinct odor and which is completely miscible with organic solvents and partly miscible with water [3,4]. Furthermore, as biobutanol has similar characteristics with gasoline, it can be used in car engines and distribution systems without any modifications [5]. The production of biobutanol over other alcohol-based compounds, such as bioethanol, is more attractive for research purposes as biobutanol has a higher energy content and lower volatility. Currently, 1-butanol is produced through a petrochemical process, and it is used as a chemical feedstock in the plastic, paints, coatings, plasticizers adhesive industries, etc. [6]. The international market demand for 1-butanol is fastened at 2.80 metric tons per year, with a market value of 4.20 billion USD. The 1-butanol market is expected to rise from 4.20 billion USD in 2017 to 5.6 billion USD by the year 2022 [7]. The main factor contributing to this forecasted increase in biobutanol demand includes a wide range of product development processes driving the market, such as latex paint formulations in industrial and architectural activities, enamels, textiles, and paper. The 1-butanol market players are especially from developed countries such as BASF, OXEA (Germany), Dow Chemical, Eastman Chemical Company (USA), PetroChina, Sinopec (China), Mitsubishi Chemical Corporation (Japan), BASF PETRONAS (Malaysia), and KH Neochem (Japan) [7]. Another reason for the growth of the 1-butanol market is increasing population, urbanization, and change in lifestyle.

The production of 1-butanol requires its separation from watery mixtures, for which the pervaporation method has been highly accepted because of its high separation and low energy consumption [8]. Poly(1-trimethylsilyl-1-propyne) (PTMSP) has been intensively studied for membrane gas separation and pervaporation [9]. PTMSP is a hydrophobic glassy polymer (*T*g > 250 °C) with an extremely high free-volume fraction (up to 25%) and exhibits microporosity [10]. The pores of PTMSP are formed during polymer solution casting, and no subsequent treatment is required. This is an advantage when compared with other membrane preparations such as immersion precipitation, cross-linking, stretching, etc. [11]. PTMSP has already been studied for the liquid separation process for the removal of ethanol, butanol, acetone, etc., from their aqueous solutions by means of pervaporation [9,12].

Polymer membranes, with an incorporated inorganic filler, so-called mixed-matrix membranes (MMMs), showed great performance in liquid separation processes [13]. The fillers can be, for example, based on silica [14] or carbon nanoparticles [15], clay [16], zeolites [17], carbon nanotubes [18], graphene oxide [19], metal-organic frameworks [20], covalent organic frameworks [21], and ionic liquids [22]. 

Recently, it has been shown that the incorporation of silica nanoparticles (SNPs) into PTMSP membranes improves their separation ability. For example, Claes et al. [23] showed that the addition of 25 wt.% hydrophobic silica into the PTMSP matrix and with a PTMSP separating layer of 2.4 μm thickness, clearly increased the permeate flux. However, this was at the expense of the alcohol/water selectivity. In the pervaporation of a 1-butanol/water mixture, a flux of 9.5 kg m^−2^ h^−1^ and accompanying separation factor of 104 was observed [23]. It has also been shown that the efficiency of mixed matrix membranes depends on the compatibility between the filler and the polymer matrix, as well as the removal of interfacial defects [24]. SNPs contain an adequate amount of silanol groups on their surface, rendering them facile for surface modification. When SNPs are modified by different organosilanes, the hydrophilic-hydrophobic properties of the SNPs can be flexibly tuned to enhance the compatibility with the polymer matrix [25].

In our previous work, we used a neat PTMSP membrane to study transient and steady-state pervaporation to remove 1-butanol from an aqueous mixture. For that purpose, a new apparatus and model allowing for the measurement of pervaporation transients and to evaluate diffusivity were developed [26]. The present work is aimed at 1-butanol/water separation using a PTMSP membrane incorporated with 5% surface-modified SNPs with a simple procedure for compactly binding with the membrane. The performance of the new hybrid PTMSP membrane is studied in relation to the total flux, selectivity, and permeability, as well as diffusivity evaluated from the pervaporation transients. Therefore, a comparison between the neat membrane (data from previously published work) and the new hybrid PTMSP membrane was conducted.

## 2. Materials and Methodology

PTMSP was purchased from Gelest, Inc. (Morrisville, Philadelphia, PA, USA), 1-butanol (min. 99%, Penta, Prague, Czech Republic), helium (4.8, Siad Czech, Prague, Czech Republic), nitrogen (4.0, Siad Czech), and liquid nitrogen (Siad Czech) were used as received. Aqueous suspension of 40 wt% silica nanoparticles (Ludox TM-40, Sigma Aldrich, St. Louis, MO, USA) was used for the incorporation in the membrane. Cationic surfactant hexadecyltrimethylammonium bromide (CTAB) (≥99%, Sigma aldrich) was used as received to modify the surface of SNPs. Physical properties of 1-butanol and water were taken from the database [27].

### 2.1. SNPs Surface Modification

**Figure 1:**
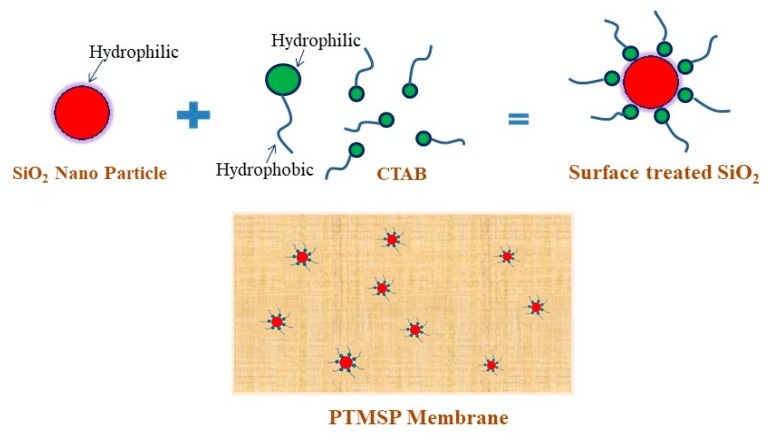
Schematic presentation for surface modification of silica nanoparticles.

Typically, 5 g of CTAB was added into 100 mL of Ludox TM-40. The resulting mixture was stirred for 20 min at 50 °C. Modified SNPs were washed with deionized water to remove excess CTAB that was not adsorbed. The resulting SNPs were dried under vacuum at ambient temperature. The processes of surface modification of SNPs was shown schematically in Figure 1. 

### 2.2. Preparation of Membrane

The mixture of 5% wt modified SNPs were blended with PTMSP using *tert*-butyl methyl ether (MTBE, min. 99.8%, Lach-ner, Neratovice, Czech Republic) via ultra-sonication for 30 min at 30% power in pulse mode (2.5 s pulse and 0.5 s pause) and stirring for 24 h using the magnetic stirrer to form a uniform suspension. The solution was then cast on a Petri dish, and the solvent was allowed to evaporate slowly over the next 48 h at room temperature. The thickness of the membrane was measured using a dial comparator (Somet, Teplické předměstí, Czech Republic). The membrane was soaked in methanol (p.a., Penta) overnight and then dried in the ambient air before its use to rejuvenate its physical structure [28]. 

### 2.3. Characterization of Membrane

#### 2.3.1. Morphology Characterization 

The surfaces of the membranes were characterized by using the scanning electron microscope (SEM) (Tescan VEGA 3-LMU, 20 kV, Brno, Czech Republic) equipped with an Energy Dispersive X-ray Spectroscopy (EDS). The samples were coated with a thin layer of gold to prevent charging.

#### 2.3.2. Contact angle Measurement

The surface hydrophobicity of the membrane was studied by measuring the static contact angle (*θ*) of the sessile water droplet using the contact angle meter (OneAttension Theta, Biolin Scientific, Stockholm, Sweden). The contact angle was determined by OneAttension 3.0 software. Before measurement, the membrane was cleaned with methanol to degrease its surface. Water droplet with a volume of 2 µL was placed on the surface of the membrane fixed to the glass substrate. The image of the water drop was captured using a high definition camera. The contact angle was defined by fitting the Young–Laplace equation around the droplet using the system software. 

#### 2.3.3. Thermogravimetric Analysis

The thermal stability of the silica filled PTMSP membranes was examined using simultaneous thermogravimetry-differential scanning calorimetry (TG-DSC). Experiments were carried out using a Setaram Sensys Evo thermal analyzer (France; operating range from −120 °C to 800 °C) equipped with a symmetrical balance and a Calvet 3D sensor. The temperature ranges from 30 °C to 800 °C was inspected with a heating rate of 10 °C/min. Nitrogen flow of 20 mL/min was used to remove corrosive gases potentially involved in the degradation and to avoid thermoxidative degradation. 

### 2.4. Pervaporation System: 

**Figure 2:**
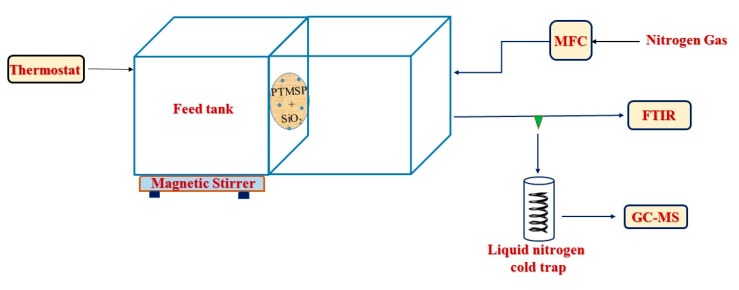
Schematic drawing of the pervaporation apparatus.

Pervaporation (PV) experiments were conducted using the previously published apparatus [26]; the schematic diagram of the system used to carry out the pervaporation experiments is shown in Figure 2. The apparatus consisted of 2 detachable cells made from duralumin. The left part of the cell (Figure 2) holds the feed solution at atmospheric pressure (average 98 kPa), and through the right part, the sweep gas (nitrogen) was conducted to the PTMSP-based membrane fixed between the 2 parts; the effective membrane area was 2.3 cm^2^. The left-side cell was provided with an inlet for temperature measurement and changing feed concentrations. All the experiments were performed at 37, 50, and 63 °C temperature with 1.5, 3.0, and 4.5 g of 1-butanol in 100 mL of water at 4 different time intervals. The feed solution was stirred during the pervaporation runs with a magnetic stirrer (250 rpm). The cells were double-jacketed and maintained at a constant temperature using a Huber Ministat 125 (Berching, Germany) water thermostat. Nitrogen was used as stripping/sweep gas with a constant flow rate of 75 STP/min by digital mass flow controller (DFC) (Aalborg, Orangeburg, NY, USA). The permeate was condensed and collected in a liquid nitrogen trap, and the permeation rate was determined from the weight of the collected samples using an Ohaus DV215CD balance (Nänikon, Switzerland) [29]. The molar fractions of 1-butanol and water in the feed and permeate were analyzed through gas chromatography.

### 2.5. Gas Chromatography Analysis

The concentration of 1-butanol in the feed and permeate was determined by gas chromatography with a polar capillary column [26]. The GC-MS system used in these studies consisted of a quadrupole instrument with a direct capillary column interface, an electron-ionization type ion source, and a quadrupole detector (Clarus 500, Perkin Elmer-Arnell, Waltham, MA, USA). Helium was used as a carrier gas at a flow rate of 0.6 mL/min, the temperature of the injector and ion source was 180 °C and 200 °C, respectively. The samples were injected with a split ratio of 1:75, and the injection volume was 0.2 µL. The mass spectrometer was operated in the electron ionization/selective-ion monitoring mode, collecting ions *m*/*z* 41 Da and 56 Da specific for butanol. The chromatograph was calibrated prior to the measurements.

### 2.6. Measurement of Transient Pervaporation for Butanol Diffusivity

Butanol diffusivity in the membrane was determined as described previously [26]. Once the 1-butanol concentration in the feed was stepwise changed, the stream of nitrogen with the permeate escaping the pervaporation unit was continuously analyzed with a FTIR spectrometer (iS10, Thermo Fisher Scientific Inc., Waltham, MA, USA) equipped with a gas cell maintained at 48 °C and a MCT/A (mercury cadmium telluride) detector. The approximate volume of the gas cell was 0.25 dm^3^ [30], and the nitrogen flow rate was 75 cm^3^ (STP) min^−1^. The intensities of selected compound-specific bands in the gaseous mixture were measured using the Omnic 8 software; 4 scans were taken for one spectrum under the resolution of 0.5 cm^−1^ in the time-series of 30 min. Bands ranging from 1146.76 to 974.88 cm^−1^ (1-butanol) were used for the analysis.

### 2.7. Measurement of Steady Pervaporation

The steady total permeate flux was determined by weighing the permeate collected over a certain time, thus

*J* = *m*/(*At*) (1)

where *m* is the weight of the collected permeate, *A* membrane area and *t* time. The separation performance of pervaporation was expressed as the separation factor

(2) $$\beta =\frac{x_{B,l}^{\;}\;/x_{W,l}^{\;}}{x_{B,0}^{\;}\;/x_{W,0}^{\;}}$$

where $x_{i,l}^{\;}$ and $x_{i,0}^{\;}$ stand for the molar fractions of the respective compounds (1-butanol, water) in the permeate and feed mixtures, respectively. 

Besides the total flux and separation factor, the solution-diffusion model is usually used to describe mass transfer in pervaporation. The flux of individual components across the membrane can be described as follows

(3) $$j_{i}=\frac{P_{i}^{.}}{l}\left(\gamma_{i_{o}}^{L}x_{i_{o}}^{L}p_{i_{o}}^{\mathrm{sat}.}-p_{i}{}_{l}\right)$$

where *j_i_* is the partial flux of component *i*, $\gamma_{i_{o}}^{L}$ is the activity coefficient of component *i* in the liquid feed (denoted by subscript 0) and $x_{i_{o}}^{L}$ is mole fraction of component *i* in the liquid feed, $p_{i_{o}}^{\mathrm{sat}.}$ is the pure component vapour pressure, $p_{i}{}_{l}$ is the partial pressure at the permeate (*l*) face of the membrane having the thickness *l* and *P_i_* is the gas permeability of the membrane for the component *i*. Since separation factor reflects not only material properties of the membrane but also of the entire experimental setup, material properties of different membranes can be well-compared using selectivity [31]:

(4) $$\alpha =\frac{P_{B}}{P_{W}}$$

The value of α naturally depends on the units used for permeability; mass-based units were used in this work. The thermodynamics of the 1-butanol solutions, which was used in Equation (3), was modeled using the NRTL model [32] with parameters taken from the literature [33].

## 3. Results 

The new hybrid PTMSP membrane had the thickness of 48 ± 2 µm. After soaking the membrane in methanol for 24 h, the membrane did not release detectable amounts of SNPs into the methanol; this was confirmed by measurement of dry membrane mass (see supplementary data). The addition of more SNPs into membrane resulted in the formation of aggregates that may perhaps cause large clusters in the top layer, and it had a high chance for defects in membranes [34]. Thus, in the present work, we used 5 wt% SNPs to cast a defect-free PTMSP membrane. 

**Figure 3:**
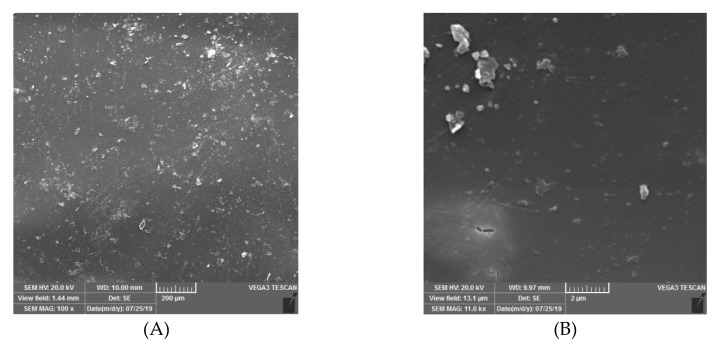
SEM images of poly(1-trimethylsilyl-1-propyne)- Silica nanoparticles (PTMSP-SNPs). (**A**) Surface of membrane (**B**) with high magnification.

The surface morphology of the PTMSP hybrid membrane containing 5 wt% of SNPs was evaluated using SEM (Figure 3). The general problem with the addition of modified silica into membranes is the formation of aggregates that could cause large clusters in the top layer [34]. It could be seen from Figure 3A that the SNPs were distributed homogeneously throughout the PTMSP matrix because of the hydrophobic interaction between PTMSP and SNPs as schematically shown in Figure 1.

**Figure 4:**
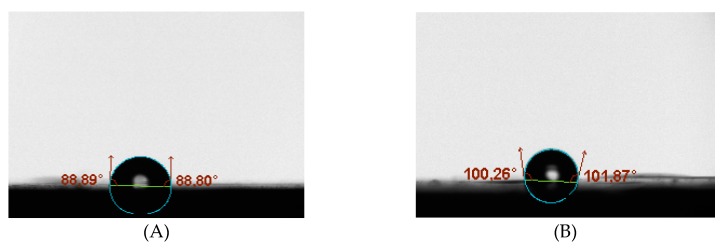
Contact angle measurement (**A**) Pure PTMSP membrane. (**B**) Hybrid membrane.

To measure the hydrophobic nature of PTMSP membranes filled with SNPs, water contact angle measurements were carried out. The so-obtained images are presented in Figure 4. It is clear that the water contact angles of the hybrid membranes were higher (*θ* = 101°) than those for the neat PTMSP membrane (*θ* = 88.85°). The likely reason for such enhanced hydrophobicity was the incorporation of the silica particles whose surface was changed from hydrophilic to hydrophobic by the use of CTAB surfactant. Besides that, this observation was probably influenced by the change of the surface roughness (Figure 3). Overall, the hydrophobicity of the membranes was clearly improved when the SNPs were introduced into PTMSP.

**Figure 5:**
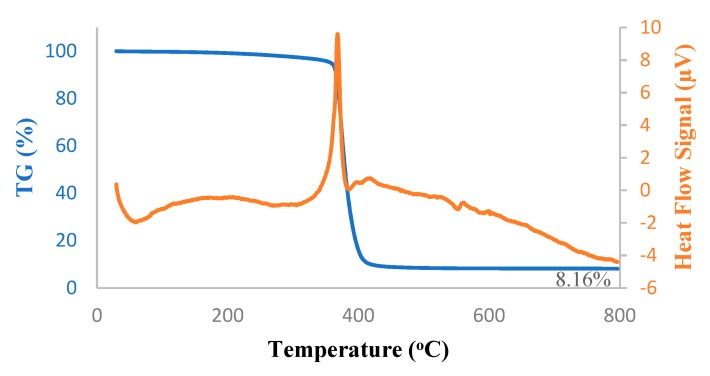
Thermogravimetry-differential scanning calorimetry (TG-DSC) thermograms of pure PTMSP membrane.

**Figure 6:**
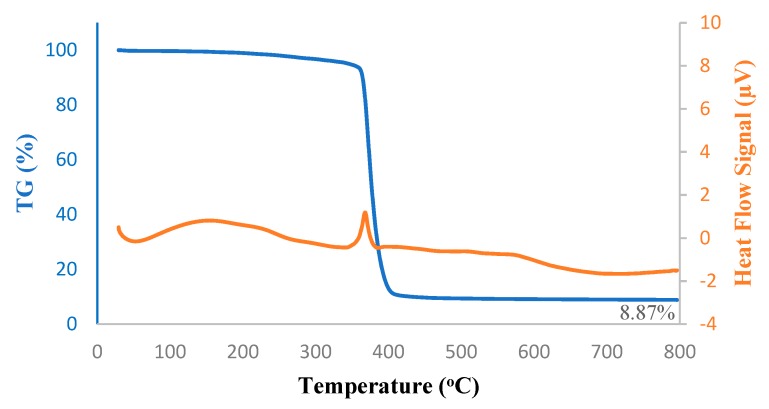
TG-DSC thermograms of PTMSP membrane blended with 5 wt% SNPs.

Thermal decomposition kinetics and stability of the hybrid PTMSP membrane, as well as that of pure PTMSP membrane, were investigated using TG-DSC under a nitrogen atmosphere; results are shown in Figure 5 and Figure 6. The melting peak onset arose steadily from 200 °C. Above 300 °C, the major decomposition-isomerization peak started and reached the peak melting point at 400 °C. At 800 °C, only 8.1 wt% of pure PTMSP and 8.8 wt% of hybrid PTMPS remained, which was in good agreement with the addition of 5 wt% SNPs to the membranes. From the DSC peaks, the melting took place at 370 °C in both pure and hybrid membranes. In the case of the hybrid membrane, the heat of this decomposition was lower due to the SNPs.

**Figure 7:**
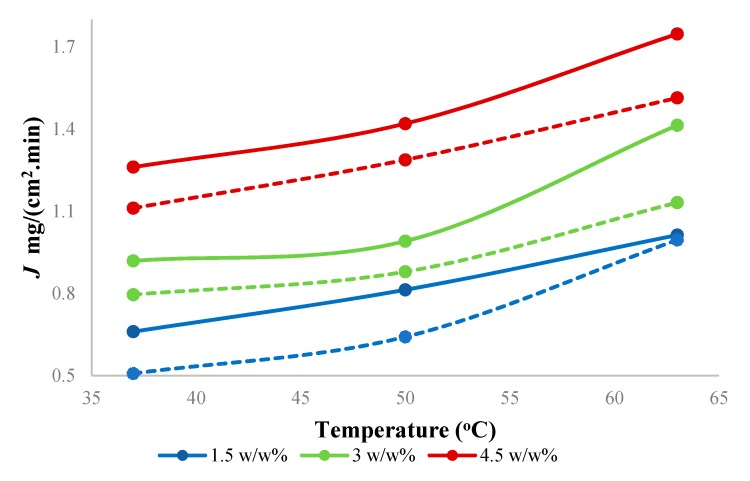
Effect of feed concentration on total flux using hybrid PTMSP membrane at different temperatures. Solid lines represent a hybrid PTMSP membrane, and dotted lines represent a neat PTMSP membrane. Data for pure PTMSP were taken from the literature [26].

Feed concentration is an important variable in the pervaporation. Figure 7 shows the effect of the feed concentration of butanol on total flux through the PTMSP membrane filled with SNPs. The total flux increased from 0.66 to 1.26 mg/(cm^2^.min) with increasing temperature from 37 to 63 °C and from 1.00 to 1.74 mg/(cm^2^.min) with increasing butanol concentration from 1.5 g to 4.5 w/w% at 63 °C. The overall total flux through the SNPs-PTMSP membrane (thickness 48 ± 2 µm) was 15% higher compared to earlier published results with neat PTMSP membrane of a comparable thickness (46 ± 2 µm). 

**Figure 8:**
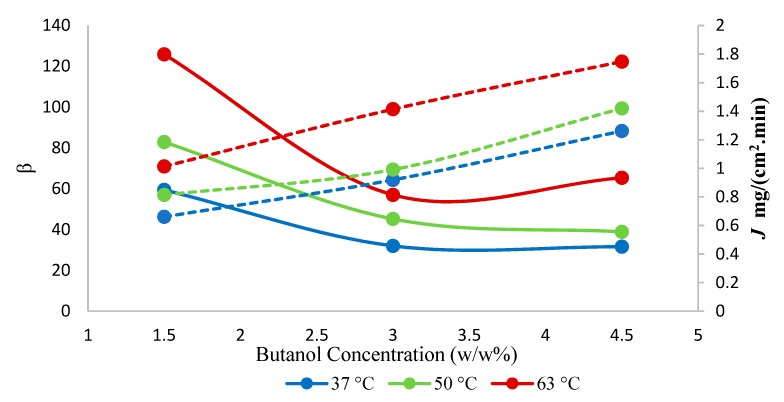
Effect of temperature on total flux (*J*) and separation factor (*β*) using hybrid PTMSP membrane. Solid lines represent separation factor, and dotted lines represent total flux.

The effect of temperature on both total flux and separation factor as a function of various 1-butanol concentrations is shown in Figure 8. The highest separation factor of 126 was observed at 63 °C at 1.5 w/w% 1-butanol concentration in the feed. Furthermore, the total flux increased with an increase in temperature. According to the solution-diffusion model, at higher temperatures, the increased difference in vapor pressure was responsible for the higher flux. As the temperature increased in feed, the change in vapor pressure was higher, which resulted in higher partial vapor pressure and provided more driving force. The apparent activation energy for permeation was calculated using a ln *J* vs. 1/*T* plot, which is a compounded parameter characterizing the overall temperature dependence of permeation flux [35]. The hybrid membrane showed lower apparent activation energy for the total flux, 14.2 kJ·mol^−1^, compared to the neat membrane 22.3 kJ·mol^−1^ (Supplementary material. Figure S1). Hence, the lowering of the apparent activation energy upon the addition of SNPs indicates the opening of the structure of the membrane. In the pervaporation processes, however, both the driving force and the permeability coefficient of a membrane for mass transport are influenced by temperature; the driving force changes as activity coefficients in Equation (3) depend on temperature [32,33]. Hence, the temperature dependence of the individual permeabilities rather than of the total fluxes are discussed below. 

**Figure 9:**
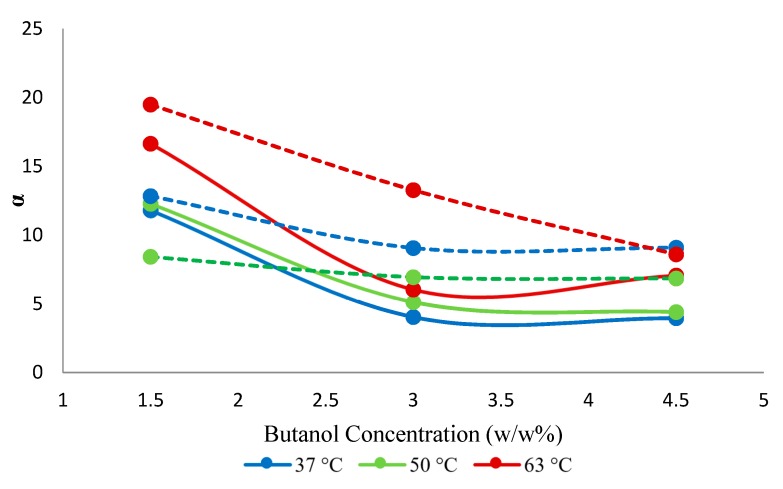
Effect of feed concentration on selectivity (*α*) against temperature. Solid lines represent hybrid PTMSP membrane and dotted lines represent a neat PTMSP membrane. Data for pure PTMSP were taken from the literature [26].

**Figure 10:**
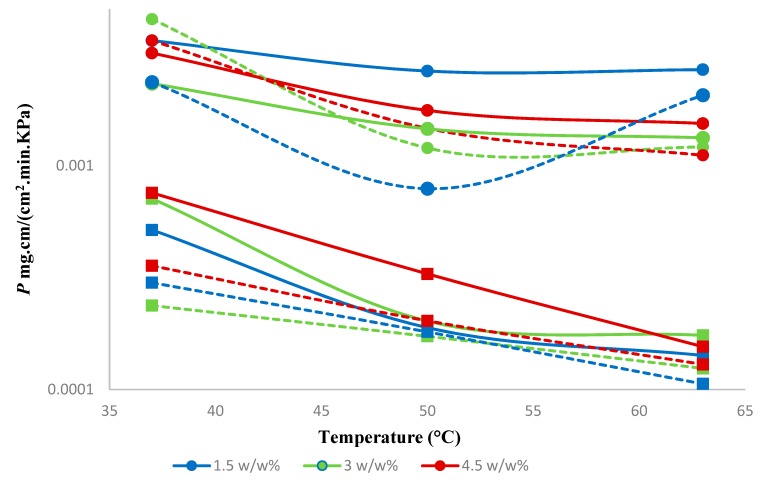
Effect of feed concentration on the permeability of 1-butanol and water. Solid lines represent a hybrid PTMSP membrane, and dotted lines represent a neat PTMSP membrane. (●—1-butanol and ■—water). Data for pure PTMSP were taken from the literature [26].

The separation factor increased by adding nanoparticles. This was presumably so due to the high adsorptive capacity of the SNPs for 1-butanol, which presumably enhanced the pervaporation separation performance of the resulting mixed-matrix membrane. The enhanced adsorption rate at the liquid/membrane interface, as a result of the contribution of the adsorptive effect of the nanoparticles, also presumably determined the increase in the butanol flux of the hybrid membranes. The increase of the selectivity with changes in the 1-butanol concentration (Figure 9) appears due to the blocking of the sorption sites of the membrane by 1-butanol, rendering the membrane less permeable towards water (Figure 10), while the membrane appears resistant against plasticization, i.e., butanol permeability does not increase significantly with the increasing butanol concentration in the feed. The maximum selectivity was observed at 63 °C at each of the three explored feed concentrations.

The permeability of both 1-butanol and water dropped noticeably with increasing temperature and increased with increasing concentration of 1-butanol in the feed (Figure 10). Negative activation energies were observed for the permeability of water and 1-butanol in both hybrid and neat membranes (Supplementary materials, Figures S2–S4), which is consistent with the literature [36]. As we show below, the activation energy of 1-butanol diffusion was positive. Hence, this evidences the exothermic dissolution of 1-butanol in PTMSP and in the hybrid SNPs-PTMSP, while the exothermic dissolution of water in these materials can be expected.

When compared to the neat PTMSP membrane, the hybrid membrane showed an increase in the permeability of 1-butanol and water by 10% and 18%, respectively. The earlier reported results for the neat PTMSP membrane [26] showed, on average, 6% higher selectivity compared to the present hybrid membrane. The hybrid membrane appears, however, more selective in the case of diluted feeds (Figure 9). Similar results were observed with silica-filled PTMSP membranes, which showed permeate flux increases, but selectivity ethanol/water remains unchanged [23].

**Figure 11:**
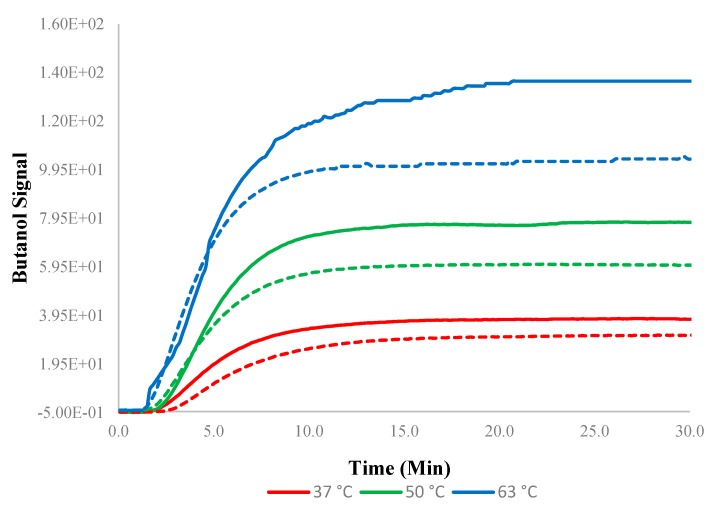
Transients for 0–1.5 w/w% at different temperatures. Solid lines indicate experimental data devised from the experiments using the PTMSP membrane blended with SNPs and dotted lines without SNPs (neat membrane). Data for pure PTMSP were taken from the literature [26].

The pervaporation transients observed for the experiments at higher temperatures were naturally more rapid (Figure 11). The addition of 5 wt% SNPs into PTMSP resulted in more rapid transients when compared to normal PTMSP membranes, especially at higher temperatures (63 °C).

**Figure 12:**
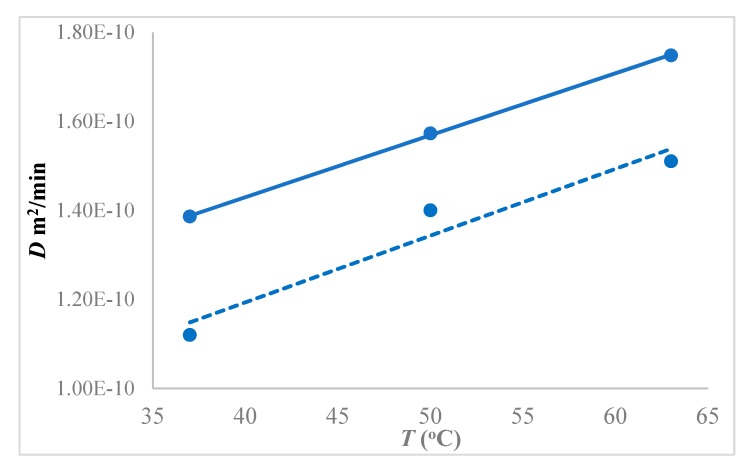
Diffusivity of 1-butanol in hybrid PTMSP-SNPs membrane at different temperatures using 1.5 w/w% feed concentration. Solid lines represent a hybrid PTMSP membrane and dotted lines neat membrane. Data for pure PTMSP were taken from the literature [26].

The diffusivity of 1-butanol in the hybrid membrane increased with increasing temperature at 1.5 w/w% feed concentration, as shown in Figure 12. The diffusivity increased by 12.2% (from 1.39 × 10^−10^ to 1.57 × 10^−10^ m^2^/min) by changing temperature from 37 °C to 50 °C, whereas it increased by 24% (from 1.39 × 10^−10^ to 1.74 × 10^−10^ m^2^/min) by changing temperature from to 37 °C to 63 °C, all for the 1.5 w/w% 1-butanol concentration in the feed. When compared to the neat membrane, the hybrid PTMSP showed 15% higher diffusivity of 1-butanol at 1.5 wt% feed concentration than pure PTMSP. 

**Figure 13:**
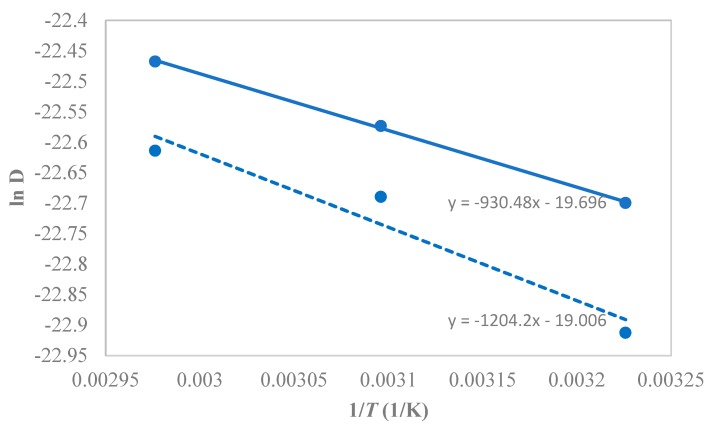
Arrhenius-type plot for the temperature dependence of the 1-butanol diffusivity in PTMSP-SNPs and neat PTMSP membranes at 1.5 w/w% feed 1-butanol concentration. The solid line represents the hybrid PTMSP-SNPs membrane and dotted lines for the neat PTMSP membrane. Data for pure PTMSP were taken from the literature [26].

The temperature dependence of the 1-butanol diffusivity in PTMSP followed the Arrhenius type of dependence, yielding, on average, the activation energy of 1-butanol diffusion of kJ·mol^−1^ (Figure 13), while 10.1 kJ·mol^−1^ for the neat PTMSP. Hence, the SNPs created additional pathways in the matrix, thereby enhancing the butanol diffusivity and permeability in the membrane.

The Pervaporation performance of different membranes in butanol-water mixtures from literature are shown in Table 1. The hybrid PTMSP membrane in this work has a total flux of 0.99 mg·cm^−2^min^−1^ with the separation factor of 126.1 at 63 °C for 1.5% wt% of 1-butanol in the feed. The total flux and separation factor for our hybrid PTMSP membrane appeared comparable or higher when compared to other membrane materials reported in the literature.

The Pervaporation performance of different membranes in butanol-water mixtures from literature are shown in Table 1. The hybrid PTMSP membrane in this work has a total flux of 0.99 mg·cm^−2^min^−1^ with the separation factor of 126.1 at 63 °C for 1.5 wt% of 1-butanol in the feed. The total flux and separation factor for our hybrid PTMSP membrane appeared comparable or higher when compared to other membrane materials reported in the literature.

## 4. Conclusions

In order to further enhance the butanol separation performance of PTMSP membranes, SNPs were incorporated into the polymer. The compatibility between the PTMSP matrix and SNPs was assured by modifying the silica nanoparticles surface with CTAB. The SEM images showed that the SNPs were compatible with the PTMSP and were uniformly distributed across the membrane. From contact angle measurement, it was observed that the hydrophobic nature of the modified membrane was increased from the normal membrane. For the modified membrane, the pervaporation measurements showed maximum separation factor of 131 at 50 °C, 3 w/w% 1-butanol and the total flux of approximately 1.74 mg·cm^−2^·min^−1^ at the higher temperature (63 °C) and 1-butanol concentration 4.5 w/w% in the feed. According to the transient data for pervaporation, it was confirmed that the butanol diffusivity was elevated significantly for the hybrid membrane when compared with the neat PTMSP membrane. Finally, the new mixed matrix PTMSP silica hybrid membrane exhibited comparatively high separation factors, similar to that of the pure PTMSP and, at the same time, showed higher permeability. Hence, these modified PTMSP membranes can be beneficial for future applications involving the separation of 1-butanol from aqueous solutions.

## Figures and Tables

**Table 1:** Comparison of different membranes using butanol-water mixtures by pervaporation process.

Membrane Type	Thickness (µm)	Temperature (°C)	Feed Concentration (Butanol)	Total Flux (g·m^−2^·h^−1^)	Separation Factor	Reference
PTMSP-silica	2.4	50	5 wt%	950	104	[23]
PTMSP/PDMSM	30	25	2 wt%	120	128	[37]
Pure PTMSP	22	25	1.5 wt%	60	55	[38]
Pure PDMS	30	55	1.5 wt%	720	34	[39]
PEBA 2533	100	23	5 wt%	32	12	[40]
PEBA with CNT (10%)	50	37	1 wt%	139	18	[41]
PDMS filled silicalite-1	19	50	1 wt%	191	111	[42]
PDMS/ceramic composite membrane	10	40	1 wt%	457	26	[43]
Reinforced PTMSP/stainless steel	40	60	1.0 wt%	560	83	[44]
BEA-type zeolite membranes	-	45	1 wt%	620	229	[45]
Our work with pure PTMSP	46	37	1.5 wt%	85	67	[26]
PTMSP-SNPs (modified silica nanoparticles)	48	37	1.5 wt%	110	59	Present work
	48	50	1.5 wt%	135	83	
	48	63	1.5 wt%	165	126	

## Supplementary Materials

The following are available online at https://www.mdpi.com/2077-0375/10/4/55/s1, Figure S1: Arrhenius-type plot for 1.5 w/w% feed 1-butanol concentration. Apparent activation energy for pervaporation: Ea, hybrid = 14.2 kJ/mol, Ea,PTMSP = 22.3 kJ/mol. Solid lines represent hybrid PTMSP membrane, and dotted lines represent neat PTMSP membrane, Figure S2: Plots illustrating the activation energy for the permeability coefficient of 1-butanol and water using 1.5 w/w% feed 1-butanol concentration. Solid lines represent hybrid PTMSP membrane and dotted lines represent neat PTMSP membrane. (■—1-butanol and ●—water), Figure S3: Plots illustrating the activation energy for permeability coefficient of 1-butanol and water using 3 w/w% feed concentration. Solid lines represent hybrid PTMSP membrane and dotted lines represent neat PTMSP membrane. (■—1-butanol and ●—water), Figure S4: Plots illustrating the activation energy for permeability coefficient of 1-butanol and water at using 4.5 w/w% feed concentration. Solid lines represent hybrid PTMSP membrane and dotted lines represent neat PTMSP membrane. (■—1-butanol and ●—water), Table 1: Activation energy for both hybrid and neat membrane at shown concentrations of 1-butanol in the feed.

## Nomenclature

*A* (m^2^)	Membrane area;.
*D* (m^2^/min)	Diffusivity
*J* (mg·cm^−2^·min^−1^)	Flux
*l* (µm)	Thickness
p (Pa)	Pressure
*P* (mg·cm·cm^−2^·min^−1^·kPa^−1^)	Permeability
*R* (J mol^−1^ K^−1^)	Universal gas constant
*T* (K or °C)	Temperature
*x*	Molar fraction

## Abbreviation

ABE	Acetone butanol ethanol
FTIR	Fourier transform infrared spectroscopy
GC-MS	Gas chromatography mass spectrometry
HPLC	High Performance Liquid Chromatography
MFC	Mass flow controller
NRTL	Non-random two-liquid model
SNPs	Silica nano particles (Surface modified with CTAB)
STP	Standard temperature and pressure (273.15 K, 101.325 kPa)Greek letters
γ	Activity coefficient
β	Separation factor
α	Selectivity
