# Supplementary material for: Selective Separation of 1-Butanol from Aqueous Solution through Pervaporation Using PTSMP-Silica Nano Hybrid Membrane
Source: Membranes (Basel). 2020 Mar 26;10(4):55. doi: 10.3390/membranes10040055 (PMC7231369; doi:10.3390/membranes10040055)
Supplement: Supplementary file 1 [file membranes-10-00055-s001.pdf]

membranes

Supplementary Material

# Selective Separation of 1-Butanol from Aqueous Solution through Pervaporation Using PTSMP-Silica Nano Hybrid Membrane

**VSSL Prasad Talluri [1,2,4,*], Aiym Tleuova [2], Seyedmehdi Hosseini [3] and Ondrej Vopicka [4]**

[1] Department of Biotechnology, University of Chemistry and Technology, Technicka 5, 166 28 Prague, Czech Republic
[2] Chemical Engineering Department, University of Chemistry and Technology, Technická 5, 166 28 Praha 6, Czech Republic; aiym.tleuova@vscht.cz
[3] Department of Metals and Corrosion Engineering, University of Chemistry and Technology Prague, 166 28 Prague, Czech Republic; hosseins@vscht.cz
[4] Department of Physical Chemistry, University of Chemistry and Technology, Technická 5, Prague 6, 166 28, Czech Republic; ondrej.vopicka@vscht.cz
* Correspondence: talluri.chowdary526@gmail.com

**Measurement of release of SNPs into the methanol:**

A clean hybrid membrane was cut into square shape with size of 2x2 cm and then was soaked in methanol for 24 hours. After soaking the membrane was dried at 50 °C for 1 hour using oven. The mass of dried membrane before and after soaking was measured using Ohaus DV215CD balance with the precision of 0.00005g and remained unchanged. The control sample of neat membrane was subjected to the same procedure and also showed no detectable weight loss.

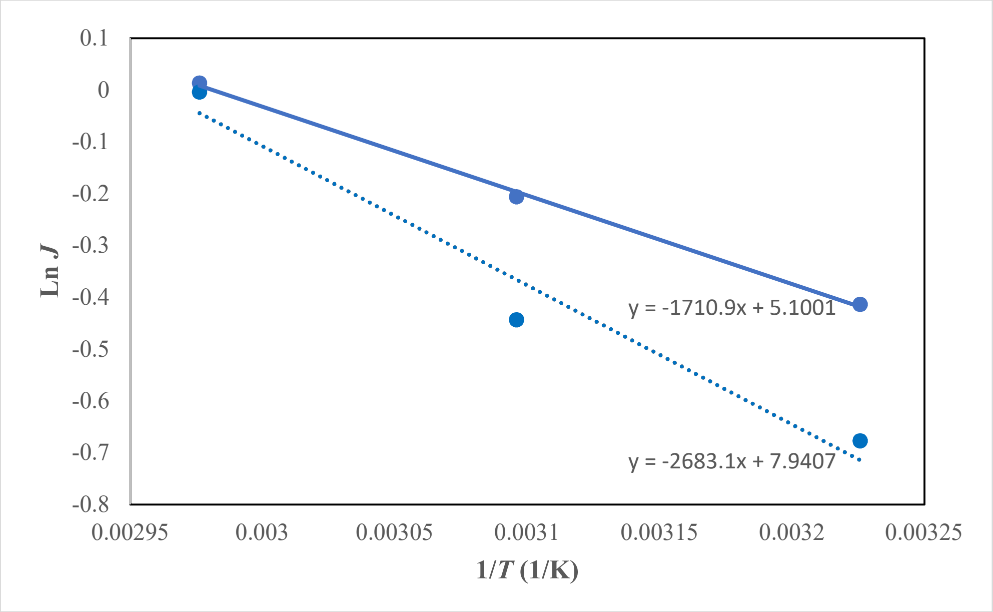


**Figure S1.** Arrhenius-type plot for for 1.5 w/w% feed 1-butanol concentration. Apparent activation energy for pervaporation: $E_{a,hybrid}$ = 14.2 kJ/mol, $E_{a,PTMSP}$ = 22.3 kJ/mol. Solid lines represent hybrid PTMSP membrane and dotted lines represent neat PTMSP membrane.

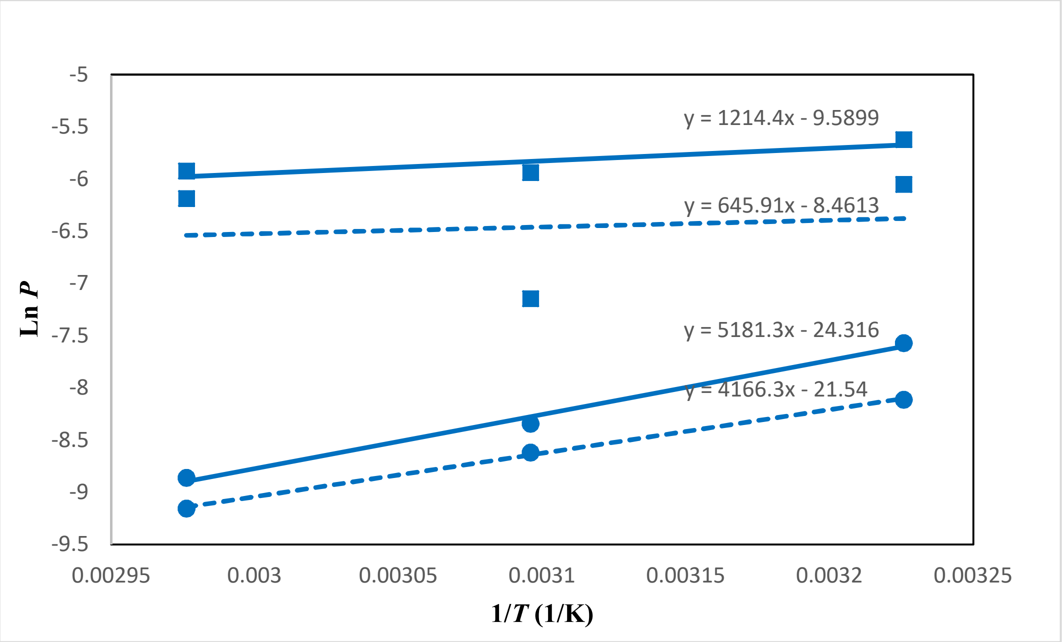


**Figure S2.** Plots illustrating the activation energy for the permeability coefficient of 1-butanol and water using 1.5 w/w% feed 1-butanol concentration. Solid lines represent hybrid PTMSP membrane and dotted lines represent neat PTMSP membrane. (■—1-butanol and ●—water).

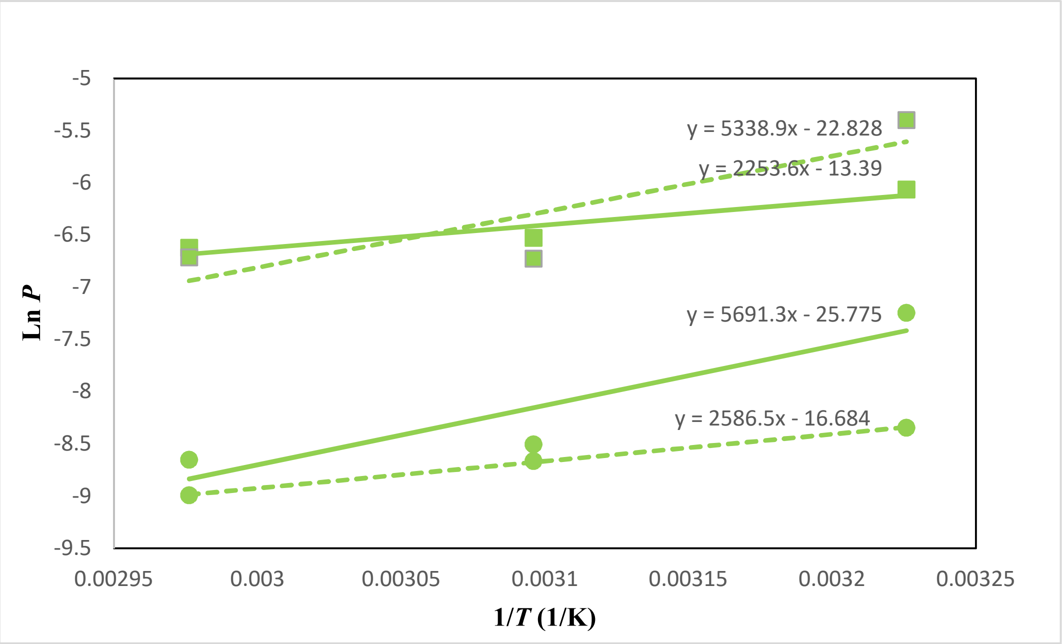


**Figure S3.** Plots illustrating the activation energy for permeability coefficient of 1-butanol and water using 3 w/w% feed concentration. Solid lines represent hybrid PTMSP membrane and dotted lines represent neat PTMSP membrane. (■—1-butanol and ●—water).

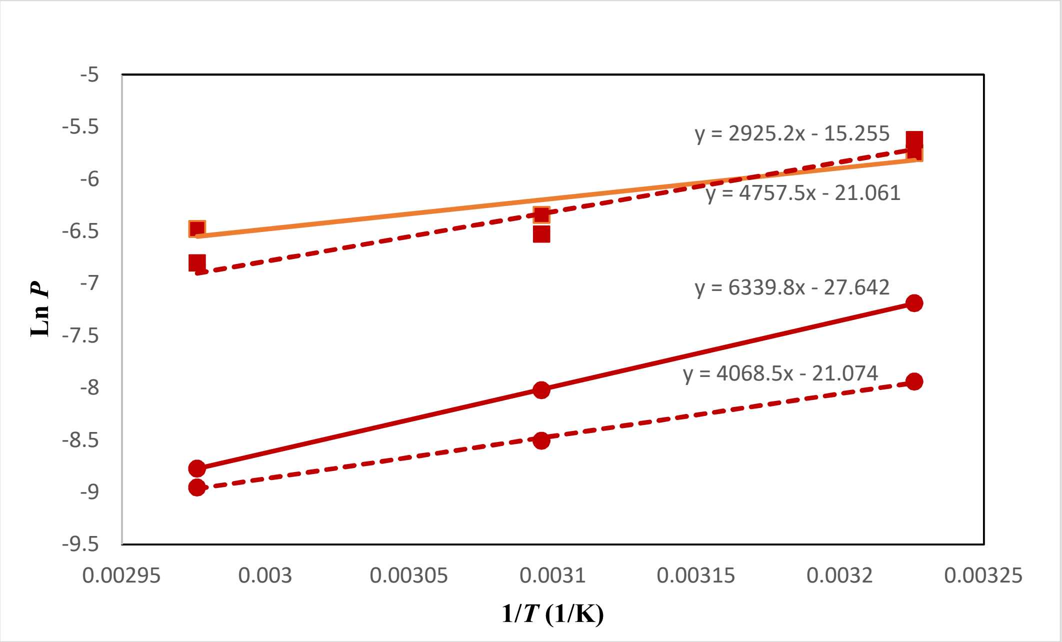


**Figure S4.** Plots illustrating the activation energy for permeability coefficient of 1-butanol and water at using 4.5 w/w% feed concentration. Solid lines represent hybrid PTMSP membrane and dotted lines represent neat PTMSP membrane. (■—1-butanol and ●—water).

**Table S1.** Activation energy for both hybrid and neat membrane at shown concentrations of 1-butanol in the feed.

| | **Hybrid Membrane (kJ·mol$^{-1}$)** | | | **Neat Membrane (kJ·mol$^{-1}$)** | | |
|---|---|---|---|---|---|---|
| | **1.5 w/w%** | **3 w/w%** | **4.5 w/w%** | **1.5 w/w%** | **3 w/w%** | **4.5 w/w%** |
| 1-Butanol | −10.1 | −44.4 | −24.3 | −44.5 | −18.7 | −39.5 |
| Water | −43.0 | −47.3 | −52.7 | −20.1 | −21.5 | −33.8 |
